# TiO_2_ and Fe_2_O_3_ Films for Photoelectrochemical Water Splitting

**DOI:** 10.3390/molecules20011046

**Published:** 2015-01-09

**Authors:** Josef Krysa, Martin Zlamal, Stepan Kment, Michaela Brunclikova, Zdenek Hubicka

**Affiliations:** 1 Department of Inorganic Technology, University of Chemistry and Technology, Prague, Technická 5, Prague 16628, Czech Republic; E-Mails: zlamalm@vscht.cz (M.Z.); brunclim@vscht.cz (M.B.); 2 Joint Laboratory of Optics, Palacky University, RCPTM, 17. listopadu 12, Olomouc 77146, Czech Republic; E-Mail: kment@fzu.cz; 3 Institute of Physics, Academy of Sciences of the Czech Republic, Na Slovance 2, Prague 14800, Czech Republic; E-Mail: hubicka@fzu.cz

**Keywords:** TiO_2_, hematite, film, sol-gel, plasmatic, photocurrent, water splitting

## Abstract

Titanium oxide (TiO_2_) and iron oxide (α-Fe_2_O_3_) hematite films have potential applications as photoanodes in electrochemical water splitting. In the present work TiO_2_ and α-Fe_2_O_3_ thin films were prepared by two methods, e.g., sol-gel and High Power Impulse Magnetron Sputtering (HiPIMS) and judged on the basis of physical properties such as crystalline structure and surface topography and functional properties such as simulated photoelectrochemical (PEC) water splitting conditions. It was revealed that the HiPIMS method already provides crystalline structures of anatase TiO_2_ and hematite Fe_2_O_3_ during the deposition, whereas to finalize the sol-gel route the as-deposited films must always be annealed to obtain the crystalline phase. Regarding the PEC activity, both TiO_2_ films show similar photocurrent density, but only when illuminated by UV light. A different situation was observed for hematite films where plasmatic films showed a tenfold enhancement of the stable photocurrent density over the sol-gel hematite films for both UV and visible irradiation. The superior properties of plasmatic films could be explained by ability to address some of the hematite drawbacks by the deposition of very thin films (25 nm) consisting of small densely packed particles and by doping with Sn.

## 1. Introduction

Thin films of titanium dioxide deposited on various supports are very useful photocatalysts in a number of applications, primarily in environmental protection. Another application is in alternative energy generation, e.g., the photoelectrochemical water splitting [1] where photogenerated holes act as an oxidant, in this particular case to evolve molecular oxygen, photogenerated electrons are transferred via an external circuit to the auxiliary electrode where they are used to evolve hydrogen.

Great advantages of TiO_2_ are its low price, high stability and nontoxicity [2]. However, for practical applications, there is a huge disadvantage consisting in a large band gap energy resulting in utilization of very small part of sunlight (4%–5%). Iron oxide (α-Fe_2_O_3_) with hematite crystalline structure has recently attracted much attention as a potentially convenient material to be used for hydrogen production via photoelectrochemical water splitting. This is due to its favourable properties such as a band gap between 2.0–2.2 eV, which allows absorbing a substantial fraction of solar spectrum, chemical stability in aqueous environment, nontoxicity, abundance and low cost. For such band gaps and assuming standard solar illumination conditions (AM 1.5 G, 100 mW·cm^−2^) the theoretical maximal solar-to-hydrogen (STH) conversion efficiency can be calculated as 15% [3]. However not everything is ideal and hematite also has certain disadvantages. Among the most cited limitations are the nonideal position of hematite’s conduction band, which is too low for spontaneous water reduction (this can be addressed by applying e.g., a PV cell to provide the additional energy needed); the low absorptivity (especially for longer wavelengths); and very short diffusion length of photogenerated holes. This creates a disaccord between the depth where charge carriers are photogenerated (in the bulk) and the distance they diffuse before recombining. Doping with elements such as Sn, Ti, Ge, Si, Nb, *etc.* can significantly increase the electronic conductivity by increasing the number of carriers. The negative effect of the short diffusion length of holes can be suppressed by using very thin films of hematite or their nanostructuring. Furthermore, remarkable overpotential of about 0.4–0.6 V for the onset of the water splitting photocurrent, which has been assigned to poor oxygen evolution kinetics on hematite surfaces and/or to the presence of surface defects acting as traps, is another crucial issue. 

The aim of this work was the preparation of TiO_2_ and α-Fe_2_O_3_ thin films of well-defined thicknesses by two methods, e.g., sol–gel and the advanced pulsed plasma deposition method of High Power Impulse Magnetron Sputtering (HiPIMS). Due to the potential applications for photoelectrochemical water splitting such prepared films were characterised by the measurement of photocurrent and open circuit potential in aqueous media containing an inert electrolyte. 

The sol-gel technique enables photocatalyst films, e.g., TiO_2_, to be prepared in a way that controls surface properties such as composition, thickness and morphology [4,5]. This technique consists of several steps: (i) precursor synthesis; (ii) precursor deposition (usually by dip-coating); (iii) drying; and (iv) calcination at elevated temperatures. Layer thickness can be controlled by viscosity of the sol-gel precursor and by the withdrawal rate during dip-coating and could be between 60–300 nm. Thicker (or multilayer) films can be obtained by repeating steps (ii), (iii) and (iv) [6]. The HiPIMS discharges are operated in pulse modulated regime with a low repetition frequency (typically about 100 Hz) and a short duty cycle (~1%) with applying high peak powers (~kW/cm^2^) during the active part of the modulation cycle [7]. A distinguishing feature of HiPIMS is its high degree of ionization of the sputtered metal and a high rate of molecular gas dissociation due to very high plasma density near the target (order of 10^13^ ions cm^−3^).

## 2. Results and Discussion

### 2.1. TiO_2_ Films

**Figure 1:**
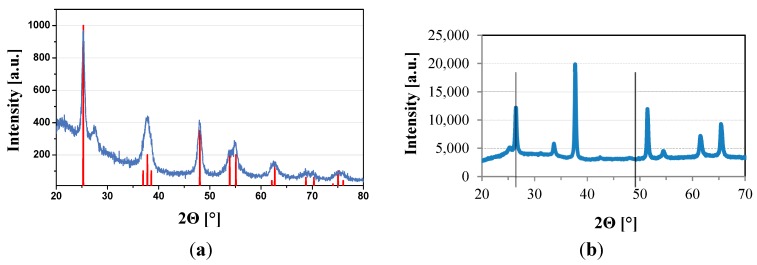
XRD patterns of (**a**) plasmatic TiO_2_ thin films deposited on quartz and (**b**) sol gel TiO_2_ thin films deposited on FTO substrate. Thickness of both films 65 nm ± 10 nm.

XRD diffraction patterns of TiO_2_ films of comparable thickness (65 nm) prepared by two different techniques are shown in Figure 1. Both types of films have an anatase crystalline structure. This is well documented in Figure 1a, where the substrate is quartz. Sol gel TiO_2_ film deposited on quartz has similar patterns so it is not shown here and instead XRD patterns of such a film deposited on FTO glass is shown as Figure 1b. It can be seen that only two Bragg diffraction peaks corresponding to anatase can be identified (due to overlapping of other bands with those corresponding to fluorine doped SnO_2_ layer), particularly for position 2Θ = 25.2° (101) and 2Θ = 48.0° (200).

**Figure 2:**
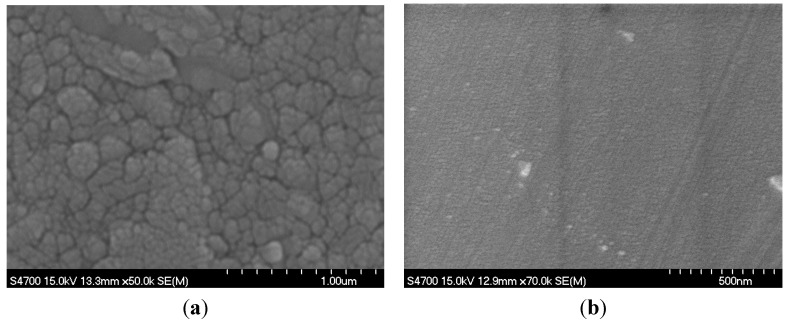
SEM surface morphology (**a**) plasmatic TiO_2_ thin films deposited on quartz and (**b**) sol gel TiO_2_ thin films deposited on FTO substrate.

The electron microscopy images (Figure 2) show that the morphologies of plasmatic (left image) and sol gel (right image) films are different. Morphology of sol gel film is smooth and due to homogeneous well transparent structure we can see only the grooves corresponding to the morphology of FTO glass substrate. On the other side the roughness of plasmatic film is significantly higher obviously due to the bigger grains.

**Figure 3:**
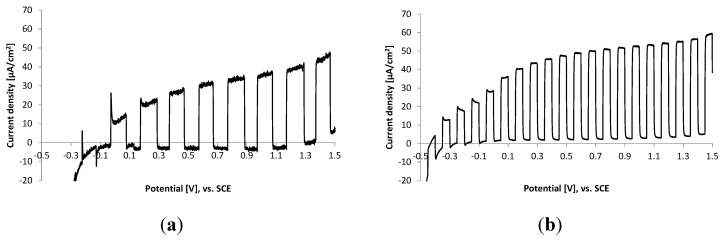
Chopped light polarization curve of (**a**) plasmatic TiO_2_ thin films and (**b**) sol gel TiO_2_ thin films. Irradiation wavelength 365 nm (1.46 mW/cm^2^), electrolyte 0.1 M Na_2_SO_4_, scan rate 5 mV·s^−1^.

Figure 3 shows the comparison of chopped light polarization curves for both types of films. The response of current to light on and light off is similar for both films, only the time interval for light on and off was two times higher for plasmatic films. 

From the shape of polarization curve, for a photocurrent equal to zero we can estimate values of OCP under light. Thus obtained values of OCP differ for both films, for plasmatic it is around −0.15 V (Ag/AgCl) while for sol gel film it is around −0.40 V (Ag/AgCl). It was reported previously (in [6]) that the value of OCP for TiO_2_ film decreased when the electrode is illuminated, which is due to the electron access generated in the nanocrystalline film. After some time of irradiation a steady state value is reached and upon turning the illumination off the photostationary potential decays and eventually reaches its initial dark value. The less negative value of OCP for plasmatic layers suggests a significantly lower amount of electrons trapped in the film upon irradiation. This can be supported by the shape of polarization curve in the vicinity of OCP where we can see fast response to light and subsequently fast decay of photocurrent resulting in very small difference between dark and light current. The possible explanation is in faster electron-hole recombination at OCP (zero current conditions) in comparison to the sol gel film.

Another difference in electrochemical properties of both films is in the potential value where the current plateau starts. For plasmatic film (even the plateau is not well developed) it starts around 0.56 V (Ag/AgCl). For sol gel films plateau starts around 0.3 V (Ag/AgCl). This indicates that for plasmatic film higher applied bias is necessary for the formation of space charge layer and sufficient electron hole separation. But when we discuss a different parameter, e.g., the difference between the open-circuit potential under light and the applied potential we can see that approximately 0.7 V is necessary for the achievement of the current plateau for both films.

### 2.2. (α-Fe_2_O_3_) Hematite Films

**Figure 4:**
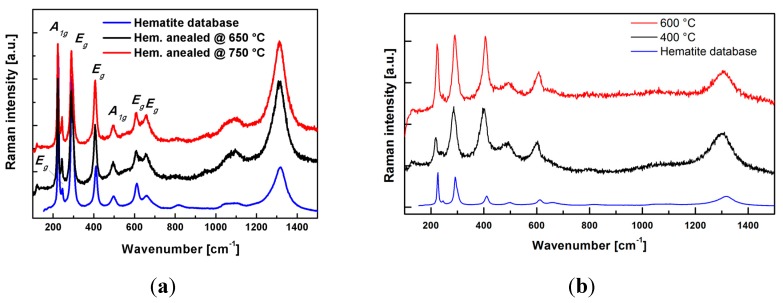
Raman spectra of (**a**) HiPIMS Fe_2_O_3_ films (thickness 40 ± 5 nm) and (**b**) sol gel Fe_2_O_3_ films (thickness 60 ± 5 nm) on FTO glass substrate.

Due to the small thickness of Fe_2_O_3_ films the crystalline structure of deposited films was assessed by Raman spectroscopy (Figure 4). We have reported previously that the hematite crystalline phase can be achieved already during the HiPIMS deposition [8]. However in order to improve the charge transfer between the hematite and FTO substrate as well as among the hematite grains themselves, the films had to be calcined at 650 and 750 °C always for 40 min in air. The database hematite spectrum is presented for reference and it unambiguously matches the measured HiPIMS Raman spectra and no significant difference (see Figure 4) between the films was evident. On the contrary to HiPIMS deposition in the case of sol gel films, the hematite structure was only achieved after calcination of the deposited precursors at 400 °C. A further increase to 600 °C has no influence on the crystalline structure.

**Figure 5:**
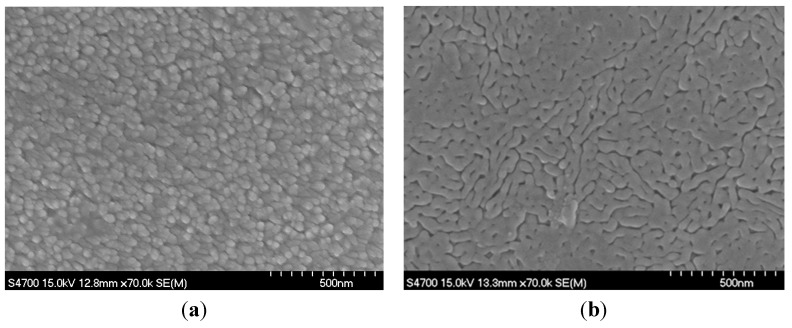
SEM surface morphology of (**a**) HiPIMS Fe_2_O_3_ films (calcined at 650 °C) and (**b**) sol gel Fe_2_O_3_ films (calcined at 600 °C) on FTO glass substrate.

With the help of SEM the surface morphology of the films was visualized (Figure 5). The morphology of sol gel films has a “worm like structure” suggesting a porous character of the film. 

On the other hand from the captured images of HiPIMS films it is clearly seen that the films consist of very small densely packed particles. Nevertheless, such organizations are a common feature of HiPIMS produced thin films in general. Furthermore the measured surface RMS roughness (AFM) was only ~2 nm for the HiPIMS films.

**Figure 6:**
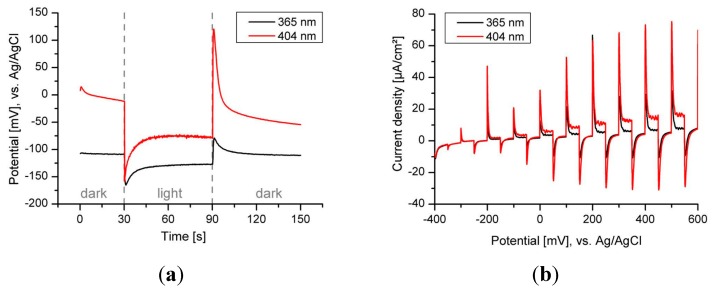
Open circuit potential (OCP) (**a**) and chopped light polarization curve (**b**) for sol gel hematite thin films calcined at 600 °C (FTO electrode). Irradiation wavelength 365 nm and 404 nm.

Sol gel hematite films calcined at 400 °C exhibit negligible electrochemical response so the data are not shown here, but the sol gel films calcined at 600 °C exhibit better performance as shown in Figure 6. The response to light is rapid and after the initial spike the photocurrent decreases due to the strong hole-electron recombination. Fast change of open circuit potential after light is switched on correlates with fast response of photocurrent to light. But the difference between the dark and light value of OCP is rather small (around −50 mV) which suggests small concentration of photogenerated electrons. Very important with respect to the PEC performance are the very sharp photocurrent spikes present in the chopped-light voltammogram when the light is turned on (see Figure 6b) denoting a high density of undesirable surface states acting as recombination centres for photogenerated carriers [9]. The surface crystalline defects and oxygen vacancies are mostly attributed to these states [10].

**Figure 7:**
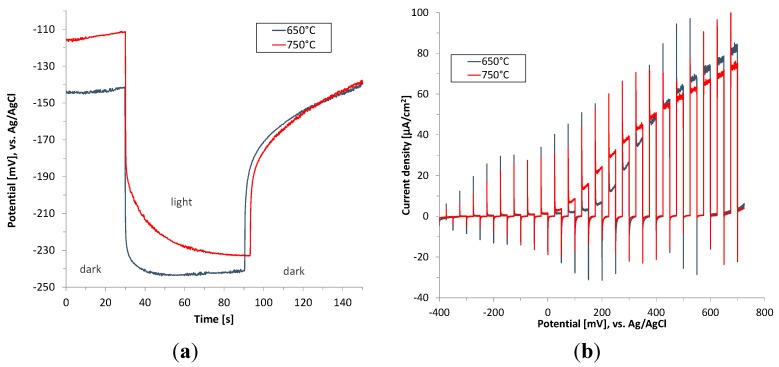
Open circuit potential (OCP) (**a**) and chopped light polarization curve (**b**) for hematite thin films calcined at 650 °C and 750 °C (FTO electrode). Irradiation wavelength 365 nm (1.46 mW/cm^2^).

**Figure 8:**
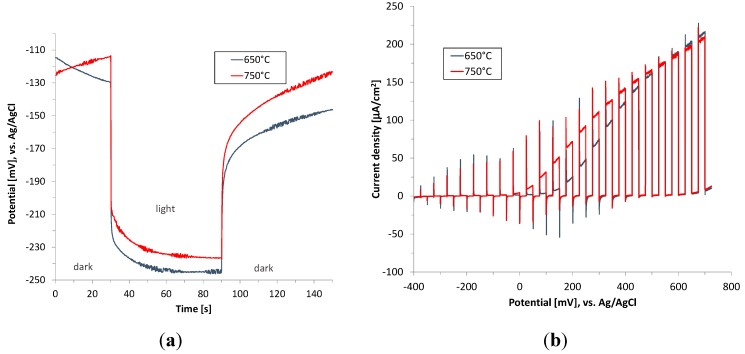
Open circuit potential (OCP) (**a**) and chopped light polarization curve (**b**) for hematite thin films calcined at 650 °C and 750 °C (FTO electrode). Irradiation wavelength 404 nm (6.1 mW/cm^2^).

Although the as-deposited plasmatic hematite films were crystalline, they exhibit almost negligible photocurrent [11]. The high density of defects and imperfections in crystalline structure, and thus a high extent of backward electron-hole pair recombination of as-deposited hematite films, is probably the main reason for the poor photoactivity. Thus, in the next step the hematite films were annealed in air at 650 and 750 °C for 40 min. and the photoelectrochemical performance of hematite films under conditions simulating UV and visible light are shown in Figure 7 and Figure 8. It was reported previously that such high temperature is needed for the diffusion of tin from the FTO substrate into hematite to occur. The diffused tin ions results in the extrinsic doping of hematite improving its electronic properties [11]. 

Increase of the annealing temperature to 750 °C does not bring about any improvement in terms of the obtained photocurrent value. On the other hand the onset of photocurrent starts already at about 0 V (*vs.* Ag/AgCl), which is shifted 100 mV negatively in relation to the film annealed at 650 °C. This phenomenon is apparently due to enhanced oxygen evolution kinetics as a consequence of self-passivating surface states serving as recombination centres. 

The observed photocurrent spikes when light is turned on and off (Figure 7b and Figure 8b) are due to the surface recombination and are typical for n-type photoelectrode [12]. The anodic current spikes have their origin in the accumulated holes at the electrode/electrolyte interface. These holes are not injected to the electrolyte as a consequence of the slow water oxidation kinetics. Instead they have ability to oxidize trap states in the bulk and on the surface. Conversely, the cathodic current spikes are generated when the light illumination is off, denoting the recombination of the accumulated holes at the semiconductor/liquid junction by the electrons diffusing from the external circuit [13,14].

One of the positive effect of HiPIMS method enhancing the photoefficiency is apparently related to a very high energy of ions and sputtered particles (which can be higher than 20 eV [15]) bombarding the substrate during the deposition by means of HiPIMS. As a consequence the interface and electrical conductivity between the FTO and hematite film is significantly boosted. A second reason for the increased photoactivity of HiPIMS is apparently attributed to the much smaller grains, which are beneficial for the process in terms of reduced electron-hole recombination due to optimal matching of nanoparticle size with the hole diffusion length [3]. As stated in the introduction the hematite films struggle with short diffusion length of the holes. This can be demonstrated on the influence of photocurrent on layer thickness where very thin film of 25 nm shows much better performance than film of thickness 40 nm [16]. The short diffusion length of the holes can be moderated not only by fabrication of very thin films but also by a careful nanostructuring in various architectures such as wormlike structures [17], nanorods [18], nanowires [19], *etc.*

### 2.3. Comparison of TiO_2_ and α-Fe_2_O_3_ Films

IPCE values for hematite and TiO_2_ films are shown in Table 1. Due to different electrolytes and thus different pH values used for photocurrent measurement, photocurrent density and IPCE were compared for the same value of potential related to RHE (1.6 V).

**Table 1:** Photocurrents of hematite and TiO_2_ films at 1.6 V (RHE).

	Wavelength [nm]	j_ph_ (at 0.98 V *vs.* Ag/AgCl)	P [10^-9^ Einstein·s·cm^2^]	IPCE [%]
TiO_2_				
sol gel	365	50.8	6.40	8.2
plasmatic	365	37.1	5.79	6.6
		**j_ph_ (at 0.57 V *vs.* Ag/AgCl)**		
Fe_2_O_3_				
sol gel	365	2.1	4.45	0.5
	404	11	20.6	0.6
plasmatic	365	74.5	4.45	17.3
	404	186.7	20.6	9.4

The measured potentials *vs*. Ag/AgCl are related to those *vs.* reversible hydrogen electrode (RHE) according to the Nernst Equation:

(1) $$E_{RHE}=E_{Ag/AgCl}+0.059\;pH+E_{Ag/AgCl}^{0}$$

where E_RHE_ is the converted potential *vs.* RHE, E^0^_Ag/AgCl_ is 0.205 V at 25 °C, and E_Ag/AgCl_ is the experimentally measured potential against Ag/AgCl reference electrode.

Unlike TiO_2_ films for hematite films we observe significant photoresponse also for the 404 nm interference filter. Owing to the band gap energy, which is tabulated to be in the region of 2.0–2.2 eV, hematite absorbs photons also in the visible range of light spectra. The overall photocurrent value at 404 nm is almost doubled comparing to the value at 365 nm but it is due to the higher incident light intensity at 404 nm (four times higher than at 365 nm). IPCE of plasmatic hematite film is in fact for 365 nm almost two times higher than for 404 nm which is due to the increased light absorption at 365 nm. Comparing the best TiO_2_ and Fe_2_O_3_ films, at 365 nm Fe_2_O_3_ has two times higher value of IPCE (17.3% *vs.* 8.2%), at 404 nm Fe_2_O_3_ reach 9.4% while IPCE for TiO_2_ was negligible.

## 3. Experimental Section

### 3.1. Glass Substrates

Two types of glass substrates were used namely quartz (SiO_2_) and fluorine doped tin oxide (FTO) transparent conducting glass (TCO) slides (TCO-7) supplied by Solaronix (Aubonne, Switzerland).

### 3.2. Preparation of TiO_2_ Films

Sol gel films were prepared using titanium(IV) isopropoxide (97%, Sigma-Aldrich, Prague, Czech Republic) as TiO_2_ precursor, absolute ethanol (p.a., Penta, Prague, Czech Republic) and ethyl acetylacetate (p.a. 99%, Fluka, Prague, Czech Republic) as solvent and nitric acid (p.a. 65%, Penta, Prague, Czech Republic) as catalyst based on the method reported previously [6,20]. Absolute ethanol was added drop wise under stirring to titanium isopropoxide. In the next step absolute ethanol was mixed with ethyl acetoacetate and nitric acid and then added to the isopropoxide mixture. Thus prepared sol was stirred under vigorous stirring for 24 h. FTO substrates were dip-coated with prepared TiO_2_ sol (withdrawal speed 60 mm·min^−1^). Deposited films were calcined at 500 °C for 1 h. 

The preparation of plasmatic films was based on reactive magnetron sputtering using a commercial Z 550 M magnetron (Leybold-Heraeus, Cologne, Germany). The diameter of the Ti target was 151 mm with purity of Ti 99.5%. The Ar-O_2_ working gases mixture with the flow rates of 20 and 10 standard cubic centimetres per minute (sccm), respectively, was used. The depositions were carried out under ambient conditions. The distance between the magnetron target and substrates was 10 cm. The films were deposited onto carefully cleaned quartz and FTO substrates. After the deposition the films were thermally treated at 450 °C for 3 h.

### 3.3. Preparation of Hematite Films

Sol gel hematite layers were manufactured as follows: the precursor was prepared by mixing Fe(NO_3_)_3_·9H_2_O, absolute ethyl alcohol and propylene oxide. The layers were deposited on the glass substrates (SiO_2_, FTO) by dip coating (withdrawal speed 60 mm/min) and after drying, calcined at 400, 500 and 600 °C for 2 h [21,22].

The HiPIMS deposition employed a metallic target of pure iron (99.995%, Plasmaterials, Livermore, CA. USA) with outer diameter 50 mm and an Ar-O_2_ atmosphere as working gases mixture in an ultra-high vacuum (UHV) reactor continuously pumped down by a turbo-molecular pump providing the base pressure of 10^−5^ Pa. Glass substrates were carefully cleaned before deposition. The working gases were fed to the reactor with the flow rates of 30 sccm and 12 sccm corresponding to argon and oxygen, respectively. The depositions were carried out at room temperature, operating pressure was 1 Pa. The pulsing frequency of DC HiPIMS discharge was in the range 70–1000 Hz with the “ON” time of 100 μs and the maximal current density achieved in a pulse was ≈5 A·cm^−2^ at 70 Hz [23].

### 3.4. Film Characterisation

The structural, morphological, electronic and optical properties of the deposited films were determined using X-ray diffraction (Seifert-XRD 3000; Panalytical HighScore Plus, Ahrensburg, Germany), space resolved Raman spectroscopy (RM 1000 Raman Microscope, Renishaw, Wotton-under-Edge, UK), AFM (Explorer, ThermoMicroscopes, Sunnyvale, CA USA) and UV-Vis absorption spectroscopy (Cary 100, Santa Clara, CA, USA).

### 3.5. Photoelectrochemical Activity Measurement

Polarization curves and the open circuit potential (OCP) measurements seem to be suitable for the assessment of the photo-electrochemical efficiency of the TiO_2_ and α-Fe_2_O_3_ thin films. Photoelectrochemical properties of films illuminated from the front side (from film/electrolyte interface) were tested in a three electrode arrangement [24,25]. The TiO_2_ or α-Fe_2_O_3_ thin film on TCO served as the working electrode, the silver-silver chloride electrode (Ag/AgCl) as the reference and a platinum sheet as the counter electrode. 0.1 M solution of Na_2_SO_4_ for TiO_2_ and 1 M NaOH for α-Fe_2_O_3_ thin films was used as an electrolyte. The exposed film area (1 cm^2^) was defined by teflon tape. The electric contact was made by pressing stainless steel to upper part of FTO layer, not covered by TiO_2_ (α-Fe_2_O_3_). The cell was connected to the optical bench (Melles Griot, Rochester, NY, USA) which included optical filters and a shutter. As the source of radiation the DC Arc polychromatic high pressure mercury lamp (LOT LSH201 Hg, Darmstadt, Germany) was employed with a characteristic broadband line source. Optical interference filters were utilized in order to ensure the layers exposure to the radiation of precise wavelength (365 ± 10 nm and 404 ± 10 nm). The photocurrents were measured using a Voltalab10 PGZ-100 potentiostat with software VoltaMaster 4 (Radiometer Analytical SAS, Villeurbanne Cedex, France). The starting potential was around the value of OCP (−0.1 V to −0.5 V (Ag/AgCl), sweeping was positive towards the end potential 1.5 V or 0.7 V (Ag/AgCl).

The incident intensity of the light when passing the filter was measured using a S1337-1010BQ photodiode (Hamamatsu Photonics K.K., Hamamatsu, Japan). The light intensity was influenced by the lamp age and thus decreased slightly during a few months period but were always in the range 1–2 mW·cm^−2^. The light intensities were always recorded before each experiment and recalculated to the incident photon flux intensity P [Einstein s^−1^·cm^−2^]. Incident photon to current conversion efficiency (IPCE) was then calculated using Equation (2):

(2) $$IPCE=\frac{j_{ph}}{F\;P}$$

where *j_ph_* denotes for the photocurrent density [A·cm^−2^], *F* is the Faraday constant (96.485 C·mol^−1^) and P is the incident light intensity [Einstein cm^−2^·s^−1^].

## 4. Conclusions

TiO_2_ and Fe_2_O_3_ represent two very promising materials for use as photoanodes for photoelectrochemical water splitting. In this study these materials were fabricated in the form of thin films by chemical sol-gel method and the plasma-assisted technique known as high power impulse magnetron sputtering (HiPIMS). The significant difference between the methods revealed here is that the plasmatic method provides the crystalline structure of anatase TiO_2_ and hematite Fe_2_O_3_ already during the deposition, whereas to finalize the sol-gel route the as-deposited films must always be annealed to obtain the crystalline phase. It has been shown that in the case of TiO_2_ films the sol-gel method produces very smooth surface of extremely small grains, while a more porous structure was observed in the case of hematite films. Slightly higher surface roughness was observed in the case of both materials fabricated by the plasmatic method.

Regarding the PEC activity of the TiO_2_ films, a higher photoresponse (about 20%) was evident for sol-gel films when illuminated by UV light using the 365 nm interference filter. A different situation was observed for the hematite films. In this case the plasmatic films showed a tenfold enhancement of the stable photocurrent density over the sol-gel hematite films. A very high concentration of the surface states acting as the recombination centres might be the reason for the poor activity of the hematite sol-gel films. The large extent of the surface states is evident as the transient spike currents of the chopped-light polarization curves. As far as the plasmatic hematite films are concerned, some of the abovementioned drawbacks for its application for the PEC water splitting were addressed in this study by deposition of very thin films of hematite (25 nm) and by doping the hematite with Sn. 
